# Quantitative Phosphoproteomics of CXCL12 (SDF-1) Signaling

**DOI:** 10.1371/journal.pone.0024918

**Published:** 2011-09-20

**Authors:** Jason A. Wojcechowskyj, Jessica Y. Lee, Steven H. Seeholzer, Robert W. Doms

**Affiliations:** 1 Department of Microbiology, University of Pennsylvania School of Medicine, Philadelphia, Pennsylvania, United States of America; 2 Protein and Proteomics Core, Children's Hospital of Philadelphia Research Institute, Philadelphia, Pennsylvania, United States of America; George Mason University, United States of America

## Abstract

CXCL12 (SDF-1) is a chemokine that binds to and signals through the seven transmembrane receptor CXCR4. The CXCL12/CXCR4 signaling axis has been implicated in both cancer metastases and human immunodeficiency virus type 1 (HIV-1) infection and a more complete understanding of CXCL12/CXCR4 signaling pathways may support efforts to develop therapeutics for these diseases. Mass spectrometry-based phosphoproteomics has emerged as an important tool in studying signaling networks in an unbiased fashion. We employed stable isotope labeling with amino acids in cell culture (SILAC) quantitative phosphoproteomics to examine the CXCL12/CXCR4 signaling axis in the human lymphoblastic CEM cell line. We quantified 4,074 unique SILAC pairs from 1,673 proteins and 89 phosphopeptides were deemed CXCL12-responsive in biological replicates. Several well established CXCL12-responsive phosphosites such as AKT (pS473) and ERK2 (pY204) were confirmed in our study. We also validated two novel CXCL12-responsive phosphosites, stathmin (pS16) and AKT1S1 (pT246) by Western blot. Pathway analysis and comparisons with other phosphoproteomic datasets revealed that genes from CXCL12-responsive phosphosites are enriched for cellular pathways such as T cell activation, epidermal growth factor and mammalian target of rapamycin (mTOR) signaling, pathways which have previously been linked to CXCL12/CXCR4 signaling. Several of the novel CXCL12-responsive phosphoproteins from our study have also been implicated with cellular migration and HIV-1 infection, thus providing an attractive list of potential targets for the development of cancer metastasis and HIV-1 therapeutics and for furthering our understanding of chemokine signaling regulation by reversible phosphorylation.

## Introduction

Chemokines are small (8–12 kDa) extracellular mediators of inflammation, chemotaxis, development and cellular survival. The C-X-C motif ligand 12 chemokine (CXCL12), also known as stromal cell-derived factor-1 (SDF-1) and pre-B-cell growth-stimulating factor (PBSF), was first cloned as a soluble factor that promoted the growth of B cell progenitors [1]. To date, two receptors for CXCL12 have been described, CXCR4 [2], [3] and more recently, CXCR7 [4]. CXCL12 and CXCR4 knockout mice are embryonic lethal [5], [6] and signaling through the CXCL12/CXCR4 axis has been implicated in organogenesis [7], [8], autoimmunity [9], [10], WHIM syndrome (Warts, Hypogammaglobulinemia, Infections, and Myelokathexis) [11], and human immunodeficiency virus -1 (HIV-1) infection [12], [13], [14]. In addition, increased CXCR4 expression has been observed in several types of cancer [15] and is frequently associated with increased metastasis and poor prognosis [16], [17], [18]. In the case of HIV-1, the viral surface glycoprotein, gp120, can act as a ligand for CXCR4-dependent signaling and influence successful infection of target cells [14], [19], [20]. A CXCR4-specific small molecule inhibitor, AMD3100 (Plerixafor), was first designed as an HIV-1 entry inhibitor, but is currently administered to mobilize hematopoietic stem cells from the bone marrow [21] and also shows promise as an anti-cancer therapeutic [22]. A greater understanding of CXCL12/CXCR4 signaling pathways may lead to more selective therapeutics for diseases such as cancer and HIV-1 infection.

The receptors for CXCL12, CXCR4 and CXCR7, are seven transmembrane receptors (7TMR) that initiate various intracellular signal transduction pathways on a variety of cell types [23]. Unlike CXCR7 [24], CXCR4 signals through a variety of heterotrimeric G proteins [23] including the pertussis toxin-sensitive Gα*_i_* upon binding either CXCL12 or HIV-1 [19], [25], [26]. Notable signal transduction pathways activated by CXCL12 in transformed and primary lymphocytes include intracellular calcium release, the mitogen-activated protein kinases (MAPK), AKT, Rho GTPases and NF-kB [27], [28], [29]. These pathways regulate basic cellular processes such as survival, migration, proliferation, cytoskeleton dynamics and gene expression [16], [29], [30].

More work is needed to understand the true breadth of CXCL12-dependent signal transduction pathways and for potential novel regulators of these pathways. This is especially important since our understanding of signal transduction pathways has changed dramatically from a simple input-output scheme to a highly interconnected network [31], [32]. With this paradigm shift, new tools and perspectives are needed to better understand signaling networks [33], [34]. Mass spectrometry-based quantitative phosphoproteomics has emerged as an important tool to examine cellular signaling events on a global, unbiased scale [35]. To date, over 40,000 phosphorylation events have been detected and catalogued, underscoring the power of this technology and pervasiveness of cellular phosphorylation [36].

In this study, we examined CXCL12/CXCR4 signaling in an unbiased fashion using mass spectrometry-based quantitative phosphoproteomics. By employing stable isotope labeling with amino acids in cell culture (SILAC) technology [37], we quantified over 4,000 unique phosphopeptides upon CXCL12 addition to CEM cells, a model human T cell line. A total of 89 phosphopeptides were deemed CXCL12-responsive, the majority of which have not been documented in CXCL12/CXCR4 signaling. Validation with various biochemical and bioinformatic analyses suggests that these CXCL12-responsive phosphosites faithfully reflect our current understanding of CXCL12/CXCR4. Our study has expanded the growing list of signaling pathways involved in CXCL12 signaling and should prove a valuable resource for future studies in areas as diverse as autoimmunity, cancer biology, and infectious diseases.

## Results

### Phosphoproteome of CEM cells

**Figure 1:**
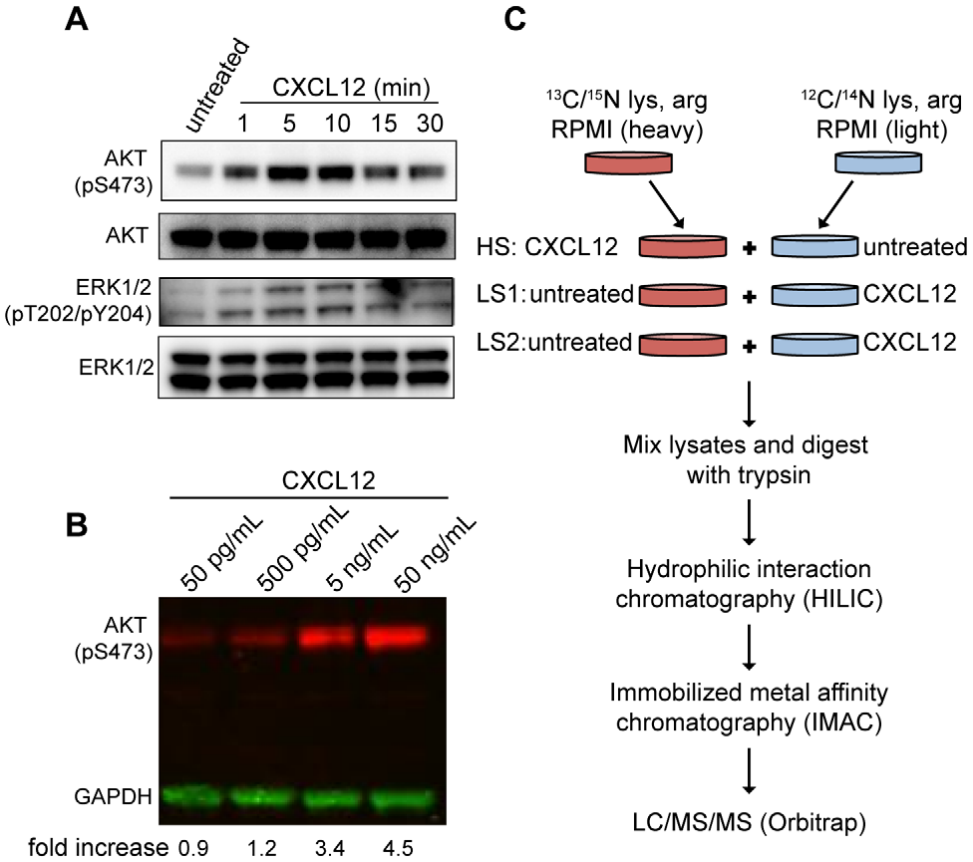
Optimization and workflow of phosphoproteomic screen. (A) CEM cells were treated with 10 ng/mL of CXCL12 for varying lengths of time and probed for AKT (pS473), total AKT, ERK1/2 (pT202/pY204), and total ERK1/2 by Western blot (see methods). (B) CEM cells were treated with varying concentrations of CXCL12 for 5 min and probed for AKT (pS473) and GAPDH by fluorescent Western blot. Band signal intensities for pAKT were normalized to GAPDH for each condition and divided by the GAPDH- normalized untreated sample. (C) SILAC heavy and light cell populations were split into three separate tubes and stimulated with 10 ng/mL CXCL12 for 5 min. Denatured lysates were combined as shown, digested with trypsin, fractionated via hydrophilic interaction chromatography (HILIC) and then immobilized metal affinity chromatography (IMAC). Individual IMAC fractions were injected into an Orbitrap XL liquid chromatography coupled tandem mass spectrometer (LC/MS/MS) and data analyzed with MaxQuant version 1.0.13.13 (see methods).

To examine CXCL12 signaling via CXCR4, we took advantage of mass spectrometry-based phosphoproteomics. We chose CEM cells, a human lymphoblastic cell line, as a model. While CXCL12 can signal through both CXCR4 and CXCR7, only CXCR4 is expressed in CEM cells [38]. To determine the kinetics and concentration dependence of CXCL12 signaling, we treated CEM cells with CXCL12 for different periods of time. Cells were lysed and analyzed by SDS-PAGE and Western blotting using phospho-specific antibodies to ERK1/2 (pT202/pY204) and AKT (pS473), both of which are phosphorylated as a result of CXCL12-CXCR4 interactions [39]. The phosphorylation of AKT and ERK1/2 peaked between 5 and 10 min following CXCL12 addition, similar to what has been observed in Jurkat cells, another lymphoblastic T cell line [39] (Figure 1A). To gauge the concentration of CXCL12 needed for maximal signaling activity, we titrated CXCL12 and measured phosphorylation of AKT at the 5 min time point. Based on this dose-response experiment, we chose to treat CEM cells with 10 ng/mL of CXCL12 for 5 min in all subsequent experiments (Figure 1B).

To quantify changes in the phosphoproteome of CXCL12-stimulated CEM cells, we took advantage of SILAC technology [37]. Cells were grown in parallel in media containing lysine and arginine labeled with the nonradioactive heavy isotopes of ^13^C and ^15^N (‘heavy’ media) or in normal media containing natural lysine and arginine isotopes (‘light’ media). The mass difference between heavy and light peptides allows for sensitive measurement by the mass spectrometer of the relative abundance of a peptide between experimental samples. Aliquots of heavy and light cultures of CEM cells were either left untreated or stimulated with 10 ng/mL of CXCL12 for 5 min at 37°C (Figure 1C). Small aliquots were taken from each experimental sample before processing to confirm CXCL12 signaling activity by probing for pERK1/2 by Western blot (data not shown). Lysates of CXCL12-treated heavy cells were mixed with lysates from untreated light cells and conversely, lysates of CXCL12-treated light cells were mixed with lysates of untreated heavy cells. One heavy-stimulated sample pair (HS) was analyzed, as were two light-stimulated sample pairs, LS1 and LS2. In addition, LS1 was split into two aliquots that were each analyzed independently by mass spectrometry providing a pair of technical replicates termed LS1a and LS1b. In our study, we define a pair of biological replicates as being HS and either LS1a, LS1b, or LS2.

**Figure 2:**
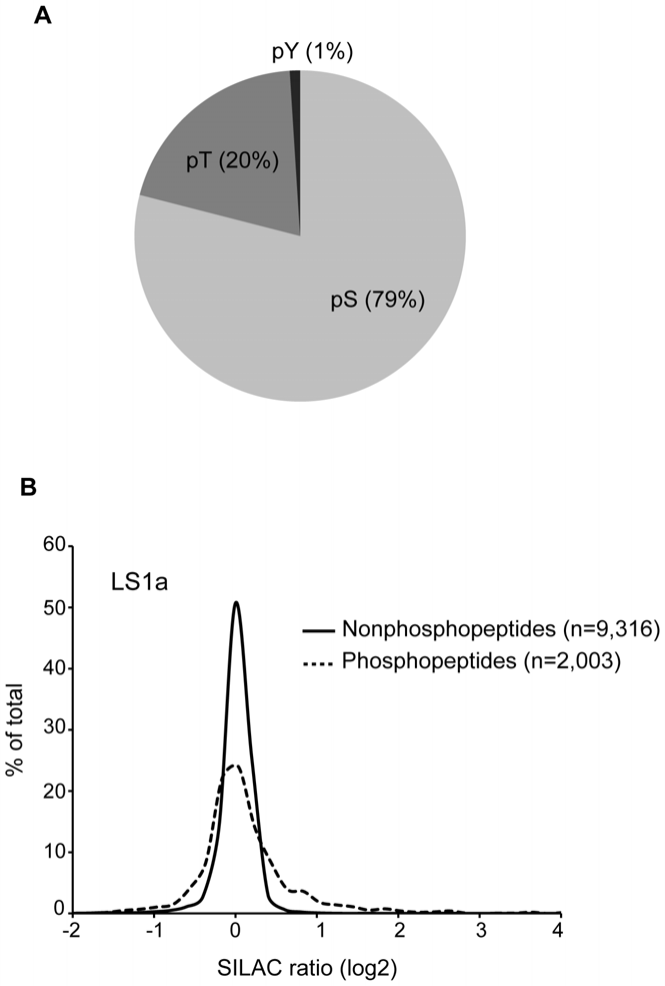
Characteristics of phosphoproteomic results. (A) When comparing the frequencies of phosphorylated serine (pS), threonine (pT) and tyrosine (pY) detected in all samples combined, pS is the predominant phosphorylated amino acid in our study. (B) Frequency distribution of SILAC ratios from phosphorylated peptides (n = 2,003) are compared to unphosphorylated peptides (n = 9,316). The LS1a sample is shown.

To resolve phosphopeptides, which are significantly lower in abundance than unphosphorylated peptides, we followed the protocol developed by McNulty and Annan [40]. Tryptic peptide mixtures were first fractionated with hydrophilic interaction chromatography (HILIC) followed by immobilized metal affinity chromatography (IMAC) to enrich for phosphopeptides (Figure 1C). From the four independent LC/MS/MS runs, we identified a total of 5,013 unique phosphopeptides from 1,780 different proteins (Table S1). 65% of these phosphosites have been cataloged in the ELM phosphosite repository [36] and in a recent phosphoproteomic study of Jurkat cells [41]. The relative stoichiometry of phosphorylated serine, threonine and tyrosine (pS∶pT∶pY) sites detected, 79∶20∶1, reflects our enrichment strategy and is consistent with similar studies [42], [43], [44] (Figure 2A). Only when a heavy and light peptide pair (a SILAC pair) within a given sample is detected by the mass spectrometer can the relative abundance of a phosphopeptide be determined. About 1,200 SILAC pairs were detected in each experiment, with an average 40.8±3.5% (mean ± standard deviation) shared between any pair of experimental samples. There was no statistical difference between the average overlaps of technical replicate and biological replicate comparisons (38.4 vs. 43.0, respectively; p = 0.2, Mann Whitney). From all four replicates, 4,074 unique SILAC pairs were identified from 1,673 proteins.

### CXCL12-regulated phosphoproteome

We established two criteria to identify phosphopeptides that were potentially regulated by CXCL12. First, the change in phosphopeptide abundance upon CXCL12 addition must be increased or decreased by ≥1.5-fold. Second, a phosphopeptide must be consistently regulated in two or more biological replicates - the HS sample and any two of the LS1a, LS1b or LS2 samples. While both heavy and light stimulations have not been routinely included in published quantitative phosphoproteomics studies, this criterion reduces potential false positives resulting merely from CXCL12-independent differences in peptide abundances between the heavy and light cells. We reasoned that phosphosites that are strongly regulated by CXCL12 would be detected in biological replicates regardless of potential variations due to heavy and light media preparations or due to biological variability.

**Figure 3:**
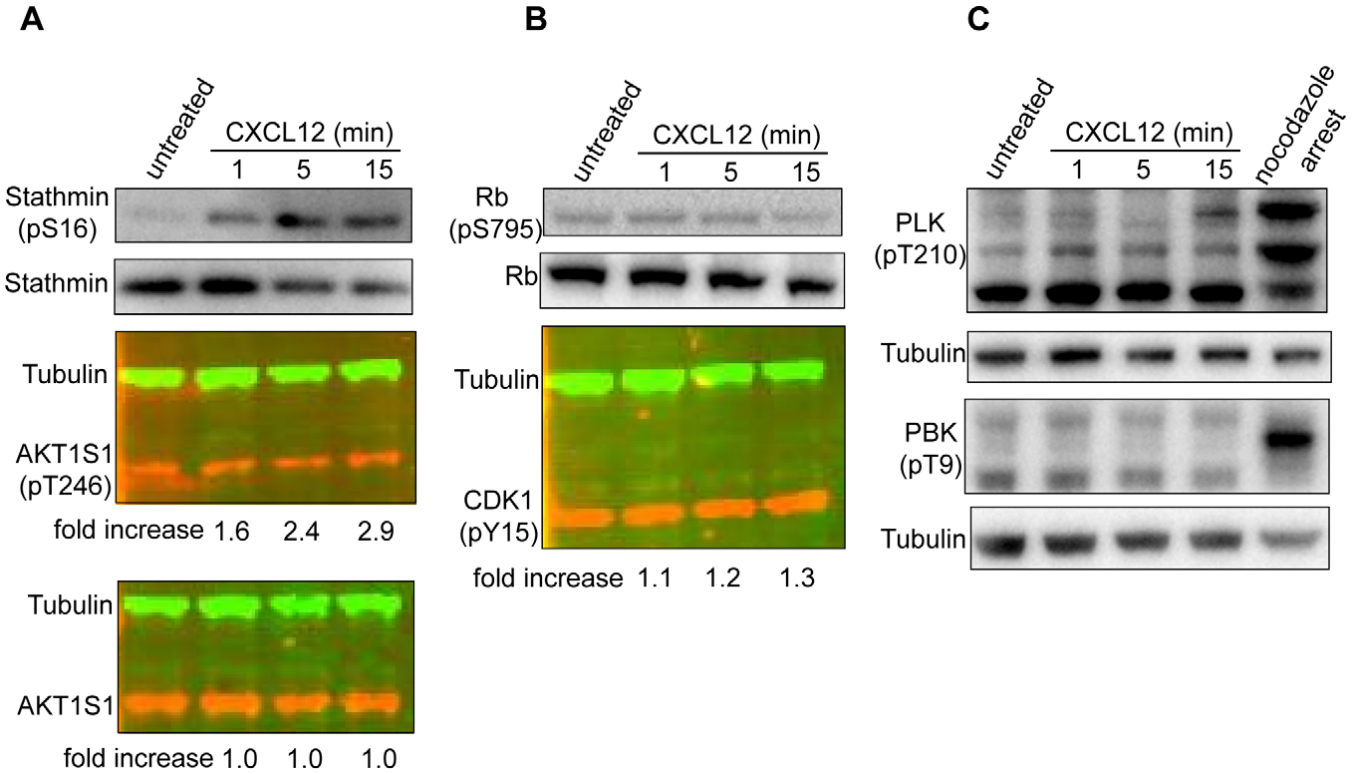
Validation of phosphosites detected by mass spectrometer. CEM cells were treated with 10 ng/mL of CXCL12 for 5 min and lysates analyzed by Western blot using antibodies for (A) stathmin (pS16), total stathmin, AKT1S1 (pT246), total AKT1S1, (B) CDK1 (pY15), tubulin, Rb (pS795), total Rb (C) PLK1 (pT210), PBK (pT9), and tubulin by Western blot. Nocodazole arrested cells were generated by treating CEM cells with 50 ng/mL nocodazole for 16 hours. Fluorescent Western blot fold-changes were calculated by normalizing their integrated intensities to tubulin for each condition, then dividing by the tublin-normalized untreated sample.

Compared to unphosphorylated peptides, 11.0±2.5 (mean ± STD) times more phosphopeptides increased in abundance ≥1.5-fold upon CXCL12 treatment, indicating a good degree of specificity (Figure 2B). This is consistent with the fact that cells were treated with CXCL12 for only 5 min - enough time for changes in phosphorylation via CXCR4-dependent signaling, but not enough time for many proteins to change in overall abundance due to either degradation or enhanced protein synthesis. Ratios of protein abundance can also be derived through quantification of several unphosphorylated peptides from the same protein. Using this approach, we found that only one out of 3,187 proteins consistently changed in abundance more than 1.5-fold in biological replicates. In contrast, 89 phosphopeptides from 81 proteins consistently changed in abundance by at least 1.5-fold between any pair of biological replicates. Mass spectrometry details of these phosphopeptides are included in Table S2. In addition, some phosphopeptides exhibited variable changes in abundance between biological replicates, increasing by ≥1.5-fold in at least one sample while decreasing by ≥1.5-fold in another, as has been reported by others [45]. Such differences may be due to CXCL12-independent differences in phosphopeptide abundance between the heavy and light cell populations, perhaps linked to the fact that these cells were propagated independently for about two weeks. To test this possibility, we treated independent cultures of CEM cells with CXCL12 as before and probed by Western blot with antibodies specific for PLK (pT210) and PBK (pT9), both of which appeared up-regulated in at least one light-stimulated sample, yet were down-regulated in the heavy-stimulated sample. As shown in Figure 3C, these phosphosites were unresponsive to CXCL12 and so may have had unequal abundances in the heavy and light cell populations.

### Validation of CXCL12-responsive phosphosites

To validate the CXCL12-responsive phosphosites, we compared them to known CXCL12-responsive phosphosites and also tested novel ones with phosphospecific antibodies by Western blot. About 50 phosphosites have been shown to be regulated by CXCL12 at different times in diverse cell types. Since we examined only a single time point in a single cell type, only a subset of these phosphosites would likely be detected in our study. Indeed, eight of these phosphopeptides were detected as SILAC pairs in biological replicates. AKT1 (pS473), ERK2 (pY204), GSK3B (pS9) and RSK1 (pS363), all known CXCL12-responsive phosphosites [28], [46], [47], surpassed the 1.5-fold change in biological replicates (Table 1). In addition, the autophosphorylation site of PAK2 (pS141) and the homologous site in PAK4 (pS181), which correlate with its kinase activity, also increased upon CXCL12 addition [48]. Two other phosphosites previously shown to be regulated by CXCL12, RPS6 (pS235, pS236), increased 1.5- fold, but in only one biological replicate and so did not meet our criteria [49].

**Table 1:** SILAC ratios of previously published CXCL12-responsive phosphosites.

				SILAC ratio (log2) per sample			
Gene symbol (alias)	Gene name	Phosphosite^a^	Sequence window	LS1a	LS1b	LS2	HS
AKT1	v-akt murine thymona viral oncogene homolog 1	473	PHFPQFpSYSASGT	ND	1.86	ND	0.95
MAPK1 (ERK2)	mitogen-activated protein kinase 1	187	TGFLTEpYVATRWY	1.79	2.15	1.75	0.63
GSK3B	glycogen synthase kinase 3 beta	9	GRPRTTpSFAESCK	0.68	0.61	0.56	0.63
RPS6KA1 (RSK1)	ribosomal protein S6 kinase, 90 kDa, polypeptide 1	372	SRTPKDpSPGIPPS	0.19	0.62	0.57	0.97
PAK2	p21 protein (Cdc42/Rac)-activated kinase 2	141	VKQKYLpSFTPPEK	0.64	0.57	1.62	1.03
PAK4	p21 protein (Cdc42/Rac)-activated kinase 4	181	RDKRPLpSGPDVGT	0.58	ND	1.01	1.27
RPS6 (S6)	ribosomal protein S6	235	AKRRRLpSSLRAST	1.45	ND	2.21	−0.39
RPS6 (S6)	ribosomal protein S6	236	KRRRLSpSLRASTS	1.38	0.22	2.16	−0.39

a Phosphosite coordinate is based off of International Protein Index database version 3.52, N.D. = not detected.

We also validated several phosphosites of SILAC pairs by probing with phospho-specific antibodies by Western blot (Figure 3). Details of these phosphosites are listed in Table 2. From independent experiments, Stathmin (pS16) and AKT1S1 (pT246), also referred to as PRAS40, both increased in abundance in CEM cells following CXCL12 addition, though neither has been previously linked to CXCL12 signaling (Figure 3A). Stathmin is a small microtubule binding protein that regulates the rates of polymerization and disassembly, or catastrophe, of microtubule chains and both phosphosites that were CXCL12-responsive, S16 and S25, have documented roles in regulating its activity [50]. AKT1S1, or PRAS40, is an Akt substrate that regulates mTOR signaling [51]. We also tested two phosphosites that were not considered CXCL12-responsive sites by our criteria, but had SILAC ratios reported in biological replicates. As expected, phosphosites from Rb (pS795) and CDK1 (pY15) showed no response to CXCL12 as determined by Western blot, making these true negatives (Figure 3C). In total, the SILAC ratios of 10/12 phosphosites were confirmed as being either up-regulated or unchanged by CXCL12 addition by independent Western blots and literature mining, consistent with the SILAC ratios determined from our experiments.

**Table 2:** SILAC ratios of phosphosites validated by Western blot.

				SILAC ratio (log2) per sample			
Gene symbol (alias)	Gene name	Phosphosite^a^	Sequence window	LS1a	LS1b	LS2	HS
STMN1	Stathmin 1	16	ELEKRApSGQAFEL	1.14	1.08	1.81	2.19
AKT1S1 (PRAS40	AKT1 substrate 1 (proline-rich)	266	PRPRLNpTSDFQKL	0.62	0.55	1.22	0.61
RB1	Retinoblastoma 1	795	PYKFPSpSPLRIPG	−0.56	0.00	−0.01	0.00
CDK1	Cyclin-dependent kinase 1	15	KIGEGpTpYGVVYKG	−0.01	−0.03	0.06	−0.09
PLK1	Polo-like kinase 1	210	DGERKKpTLCGTPN	ND	3.21	ND	−2.68
PBK (TOPK)	PDZ binding kinase	9	GISNFKpTPSKLSE	1.80	1.96	2.20	−2.48

a Phosphosite coordinate is based off of International Protein Index database version 3.52, N.D. = not detected.

### Cellular pathways involved in CXCL12 signaling

**Figure 4:**
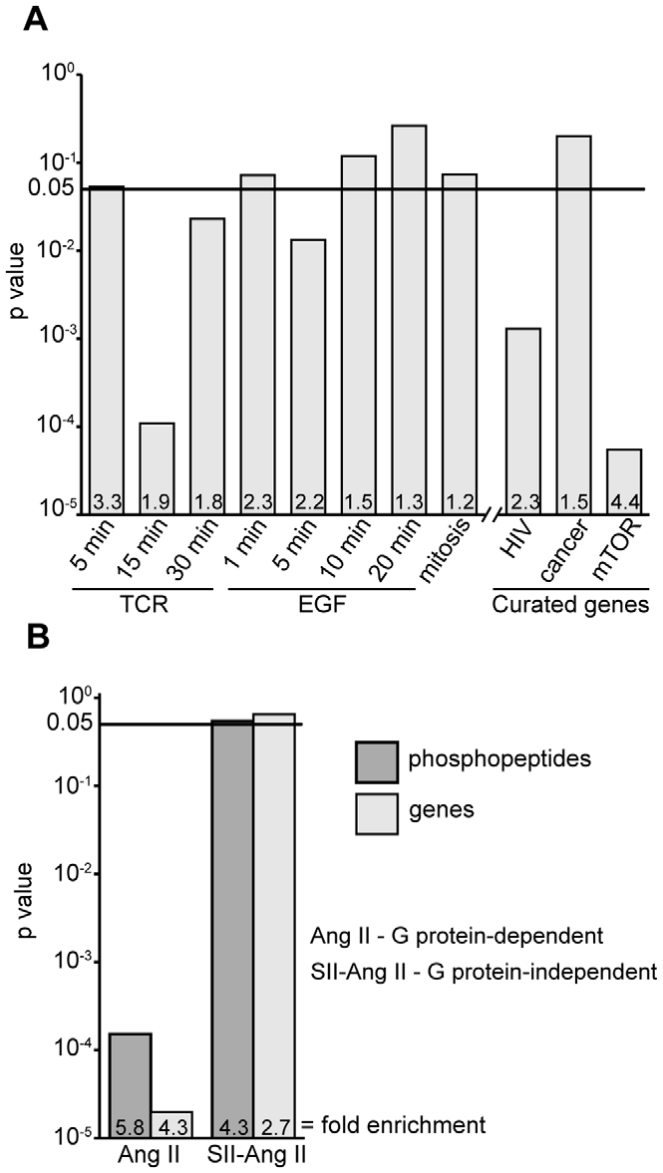
Comparison of CXCL12-responsive phosphopeptides and corresponding genes to published phosphoproteomic and manually curated datasets. Genes from various phosphoproteomic datasets and manually curated databases were compared to genes from CXCL12-responsive phosphosites using contingency tables (see methods). Fold-enrichments are listed at the base of each bar. Benjamini and Hochberg corrected p-values (Fisher's exact test) are plotted with p<0.05 considered statistically significant (solid line). (A) Genes from CD3-responsive phosphosites (TCR) [41], epidermal growth factor (EGF)-responsive phosphosites [42], and mitotic phosphosites [68] were compiled as published for the indicated time points. TCR (15 min) and EGF (5 min) are the most statistically significant, suggesting crosstalk between pathways. Unlike cancer-related genes [69], only HIV ‘interactors’ (http://www.ncbi.nlm.nih.gov/RefSeq/HIVInteractions) and mammalian target of rapamycin (mTOR) signaling genes [57] were statistically significant. (B) Phosphopeptides and corresponding genes of G protein-dependent (Ang II) and independent (SII-Ang II) signaling (3 min) were reanalyzed to reflect our experimental conditions and analysis criteria (see methods) [44]. Only the overlap of Ang II-responsive phosphosites and corresponding genes were statistically significant.

Since phosphospecific antibodies do not exist for the majority of the CXCL12-responsive phosphosites, we sought to validate our dataset at the level of cellular pathways. We compared the corresponding genes of each CXCL12-responsive phosphosite to the Kyoto Encyclopedia of Genes and Genomes (KEGG) curated pathway database [52]. Table 3 lists the ten most significantly enriched pathways. While T cell receptor signaling (hsa04660) and ErbB (hsa04012) were the only pathways from the KEGG database that achieved a statistically significant degree of enrichment, the majority of the enriched pathways identified in our screen have also been previously implicated in CXCL12 signaling, including both the mammalian target of rapamycin (mTOR) and MAPK signaling pathways [27], [53]. In addition, CXCL12 can provide costimulatory signals during T cell activation [47], [54] and transactivate HER-2 (ErbB-2) signaling in various cancer cells [55], [56]. To further test these associations, we calculated the enrichment, or overlap, of genes from two recent phosphoproteomic studies examining T cell receptor (TCR) and epidermal growth factor (EGF) signaling within genes from CXCL12-responsive phosphosites [41], [42]. Mayya et al. examined the phosphoproteome following TCR signaling that was induced by CD3 cross-linking after 5, 15 or 60 min [41]. We found a significant enrichment of genes from CD3-responisve phosphosites (15 min time point) within genes from CXCL12-responsive phosphosites (p = 3.2×10^−5^). Interestingly, overlap with the earlier (5 min) and later (60 min) time points were much less significant, suggesting kinetic specificity (Figure 4A). A similar kinetic association was seen with the EGF study reported by Olsen et al., who examined the phosphoproteome 1, 5, 10 and 20 min following the addition of EGF to HeLa cells [42]. There was a significant enrichment of hits from the 5 min EGF time point (p = 0.013), yet the 1, 10 and 20 min EGF time points were not significant. While the enrichment of the mTOR signaling KEGG pathway (hsa04150) was not statistically significant, the enrichment of genes from a manually curated mTOR signaling network [57] was highly significant in our dataset (p = 5.5×10^−5^).

**Table 3:** Enriched cellular pathways in CXCL12-responsive phosphosites.

Term	p-value^a^
hsa04660:T cell receptor signaling pathway	3.22×10^−4^
hsa04012:ErbB signaling pathway	0.0078
hsa04150:mTOR signaling pathway	0.11
hsa04010:MAPK signaling pathway	0.12
hsa05211:Renal cell carcinoma	0.14
hsa04662:B cell receptor signaling pathway	0.14
hsa04666:Fc gamma R-mediated phagocytosis	0.22
hsa04722:Neurotrophin signaling pathway	0.29
hsa04510:Focal adhesion	0.31
hsa05213:Endometrial cancer	0.31

a Benjamini and Hochberg corrected.

Enriched Kyoto Encyclopedia of Genes and Genomes (KEGG) pathways [52] in genes from CXCL12-responsive phosphosites were calculated in DAVID [93], [94].

### CXCL12 and G protein signaling

CXCL12 activates G protein-dependent and beta-arrestin-dependent signaling via CXCR4 [23]. To determine if the CXCL12-responsive phosphosites we identified are consistent with an involvement in G protein-dependent signaling, we measured the overlap of our dataset with a recently published dataset of G protein-dependent and independent phosphosites [44]. Christensen et al. stimulated 293T cells with angiotensin II (Ang II) or [Sar^1^, Ile^4^, Ile^8^] angiotensin II (SII-Ang II), both of which bind to and signal through the angiotensin II type 1 receptor (AT1R) [44]. Upon engagement of AT1R, Ang II activates and signals through G proteins, while binding of SII-Ang II does not. We parsed the data from Chrisensen et al. to more closely reflect the kinetics of our experimental design and the definition of our hit threshold (see methods) and measured the overlap of these phosphosites with the CXCL12-responsive phosphosites and corresponding genes. The overlap of Ang II-responsive phosphosites and genes was highly significant (p = 1.5×10^−5^, p = 2.0×10^−6^ respectively) compared to SII-Ang II-responsive phosphosites (p = 0.054, 0.06 respectively) (Figure 4B). Since the overlap of CXCL12-responsive phosphosites and corresponding genes with the G protein-dependent phosphosites identified by Chrisensen was highly significant, we conclude that our dataset faithfully reflects G protein-dependent CXCL12-mediated signal transduction pathways.

## Discussion

In the present study, we successfully measured the fold-change in abundance of 4,074 phosphopeptides CEM cells, 89 of which consistently changed in abundance upon CXCL12 addition in biological replicates. Several independent lines of evidence suggest that these CXCL12-responsive phosphosites faithfully reflect CXCL12 signaling events. First, Western blot analysis of several phosphoproteins confirmed their respective SILAC ratios. Stathmin (pS16) and AKT1S1 (pT246), both novel CXCL12-responsive phosphosites, increased in abundance upon CXCL12 treatment (Figure 3A). In contrast, Rb (pS795) and CDK1 (pY15) did not change when analyzed by Western blot, confirming the SILAC ratios (Figure 3B, Table 2). Second, cellular pathways that are known to be involved in CXCL12 signaling were significantly enriched in our dataset. These include T cell activation, EGF signaling and mTOR (Table 3). We were further able to confirm these associations by measuring the overlap of genes from other published independent phosphoproteomic datasets examining these pathways (Figure 4A). Lastly, a comparison of our dataset with phosphosites from a recently published phosphoproteomic study examining G protein biased ligands confirms that CXCL12-responsive phosphosites reflect G protein-dependent signaling [23], [44], (Figure 4B). Biochemical and bioinformatic analyses complemented with literature mining all reinforce the association of our dataset with known CXCL12-dependent signaling pathways.

**Figure 5:**
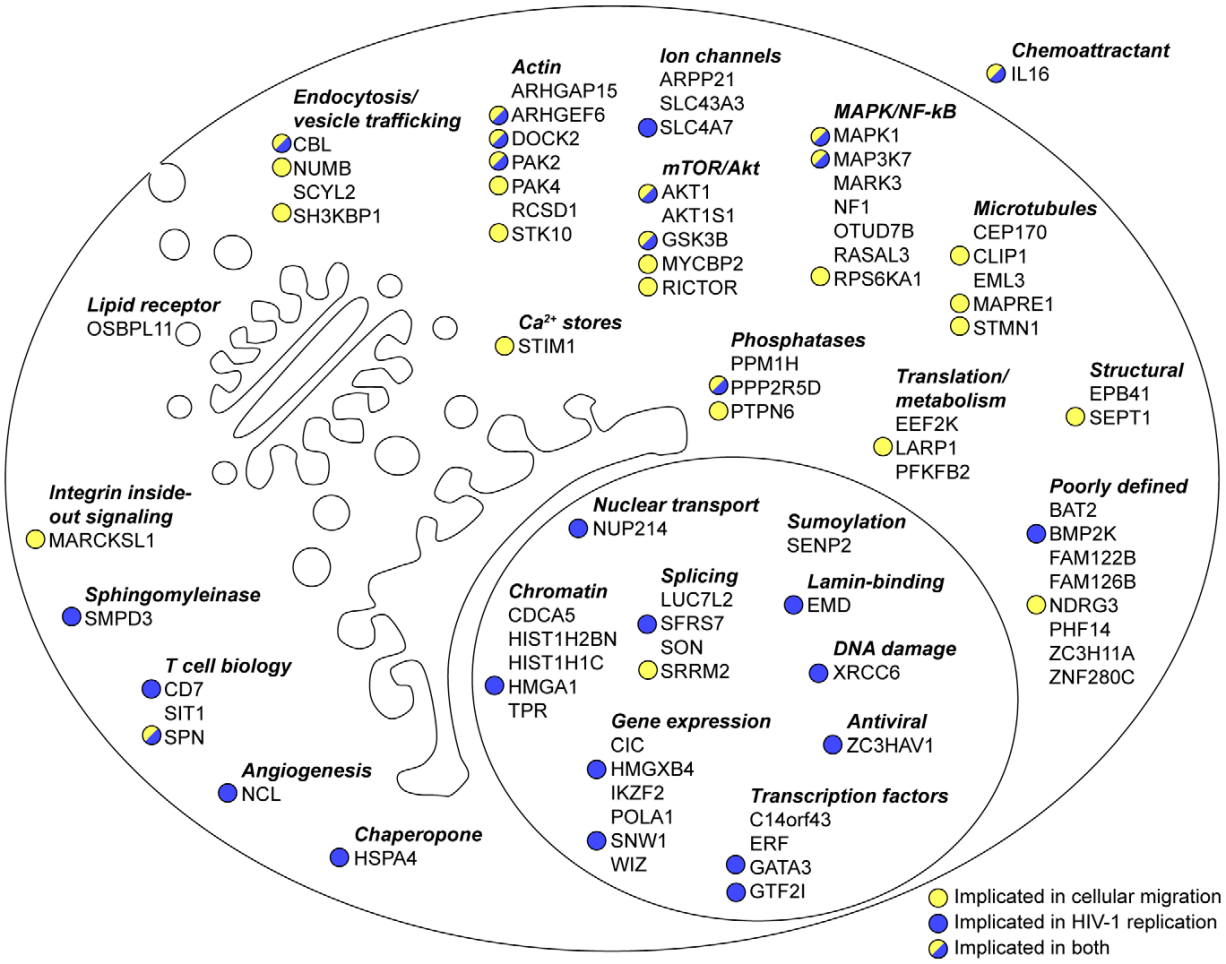
Compilation of genes from CXCL12-responsive phosphosites. Putative cellular localizations and functions of each gene are indicated as described in the literature. While some genes belong to multiple functional categories, only common associations are listed. Genes are marked with a colored circle where published experimental evidence exists to link a particular gene to HIV infection (blue), cellular migration (yellow) or both.

The results from this study expand not only the ever-growing list of catalogued phosphosites - over 1,700 of the phosphopeptides we detected are novel – but also provide a unique resource for the advancing the study of biological processes that are regulated by CXCR4. Only 34 of the 81 genes from our dataset are annotated in the KEGG pathway database, thus providing an opportunity to expand our understanding of these cellular pathway with the knowledge that a given gene product is differentially phosphophorylated by CXCL12. In addition, potential functions for a majority of CXCL12-regulated phosphosites have not yet been defined. Figure 5 shows all genes from CXCL12-responsive phosphosites according to published putative functions.

Our results also highlight an important consideration when designing SILAC experiments. PLK (pT210) and PBK (pT9) decreased ≥1.5-fold in the heavy-treated cells yet increased ≥1.5-fold in at least one of the light treated samples (Table 2). The most likely explanation for these inverted ratios is that these phosphosites are not CXCL12-responsive and had unequal abundances in the heavy and light cell mixtures (Figure 3C). To control for inherent differences in phosphosite abundance between heavy and light cultures that would otherwise contribute to false positives, one can either include a mixture of untreated heavy and light populations or stimulate each and combine with the untreated pair (Figure 1C). The latter strategy is often referred to as a ‘swap’ or ‘reverse labeling’ and phosphopeptides are only considered if the heavy- and light-treated SILAC ratios are not inverted. An added benefit to this approach is that a genuine biological replicate is obtained concurrently [58]. A drawback is that inverted heavy and light ratios can be due to either inherently different phosphopeptide abundances or biological noise. When comparing pairs of light-treated samples, LS1a, LS1b, and LS2, very few (0.08–0.4%) of each pair's SILAC ratios (≥1.5-fold) were inverted, suggesting that biological noise was not a major factor.

One of the phosphosites we validated in our screen, stathmin (pS16), has not been previously shown to be regulated by CXCL12. Stathmin is a small microtubule binding protein that regulates microtubules dynamics [50]. While few studies have examined the regulation of microtubules by chemokines, it is known that CXCL12 leads to the microtubule-dependent polarization of CEM cells [59]. Indeed, several other genes from CXCL12-responsive phosphosites identified in our study are known to regulate the microtubule cytoskeleton, including CLIP-170 (pS348), EB1 (pS165), CEP170 (pS496) and EML3 (pS176), none of which have previously been shown to be influenced by CXCL12 signaling [60], [61], [62], [63]. CLIP-170 (pS348) is found in a region of the protein with a relative high density of phosphosites, many of which can regulate its association with microtubules [64]. While the role of the human EB1 (pS165) phosphosite has not been elucidated, a small cluster of phosphosites in the analogous linker region of the yeast homolog, Bim1p, was recently shown to regulate the association of Bim1p with microtubules [65]. Taken together, our data indicate that CXCL12 signaling via CXCR4 leads to rapid phosphorylation of a number of proteins involved in microtubule dynamics, suggesting a direct role in regulating the cellular cytoskeleton for subsequent chemotaxis or some other functional response.

CXCL12/CXCR4 signaling has become increasingly implicated with various human cancers. In various types of tumors, CXCL12 can promote migration, cellular survival, and at times, proliferation [17], [66]. Genes from CXCL12-responsive phosphosites that have been linked to cellular migration are indicated in Figure 5. Consistent with previous reports [47], [67], we also did not observe increases in CDK1 (pY15) or H3 (pS10) phosphorylation, both hallmarks of cell cycle progression, upon CXCL12 addition to CEM cells at the time point studied (Figure 3B, data not shown). Also, genes from M-phase responsive phosphopeptides were not enriched in our dataset (p = 0.073) nor were genes from a manually curated collection of cancer related genes [68], [69] (p = 0.20) (Figure 4A). The mTOR pathway has been linked to cancer cell migration and our data support a model of signal cross-talk between CXCR4 and mTOR during cancer cell metastasis [53], [70] (Figure 4A). For example, recent work has shown that RICTOR, a member of the mTOR pathway, is required for neutrophil chemotaxis yet a connection between RICTOR and CXCR4 signaling has not been documented [71]. From our dataset, RICTOR contains two CXCL12-responsive phosphosites, pS1282 and pS1302. While the function of these phosphosites during CXCL12 chemotaxis has not been documented, these phosphosites may regulate RICTOR-dependent chemotaxis.

CXCR4 is also a coreceptor for human immunodeficiency virus-1 (HIV-1) and an important focus of research in HIV-1 biology [13], [14], [72]. In support of this, genes from a curated functional HIV interaction database from NCBI were significantly enriched in CXCL12 hits (p = 4.4×10^−4^) (Figure 4A). In total, 23 of the 78 genes in our dataset have a documented role in HIV infection (Figure 5), several of which have diverse roles in HIV infection. For example, BMP2K has been shown to be important for early steps in HIV infection [73], HMGA1 and XRCC6 have been implicated in HIV integration into host genomic DNA [74], [75] and GATA3, SFRS7, SNW1 have all been linked to HIV gene expression [76], [77], [78]. Little is known of the roles of phosphorylation or CXCR4 signaling on the activity of these proteins, yet HIV may modulate the activity of these proteins in a CXCR4-dependent manner to facilitate infection of the target cell. This is certainly plausible since it has already been demonstrated that cellular signals transduced by HIV during entry can affect multiple stages of the HIV lifecycle [79], [80], [81]. A growing body of work has also implicated CXCR4 and various chemokines in regulating infection of resting CD4^+^ T cells [80], [82], [83], [84], an important latent reservoir of HIV in infected individuals [85]. Therefore, it's plausible that CXCL12-responsive phosphoproteins from our study may regulate entry and integration of resting CD4^+^ T cells. Future studies can also address potential differences between signaling of CXCR4 and CCR5, another HIV-1 coreceptor, since differential engagement of these chemokine receptors can have unique effects on target gene expression [86] and host factor requirements for infection of primary cells [87]. In all, our study uncovered several members of signal transduction pathways that HIV-1 may modulate in order to successfully infect T cells, of which targeting has become an attractive avenue for anti-HIV-1 therapeutics [88].

O'Hayre et al. recently published a study examining the phosphoproteome of CXCL12-treated primary chronic lymphocytic leukemia cells, a cancer of B cells [89]. Our studies differ in target cell (B vs. T cells), phosphopeptide enrichment strategy (IMAC alone vs. HILIC and IMAC), and method of quantification (semi-quantitative label-free spectral counting vs. SILAC). Of the 13 phosphoproteins reported by O'Hayre et al. to have spectral counts suggestive of CXCL12-responsiveness, only half were detected as SILAC pairs by at least one phosphopeptide in our analysis, yet none were considered CXCL12-responsive. Interestingly, both of our studies detected novel CXCL12-responsive AKT substrates, PDCD4 (pS457) [89] and AKT1S1 (pT246) (Figure 3A), underscoring the potential role of AKT signaling and leukemias [90].

Phosphoproteomic examination of signaling pathways is poised to greatly advance signal transduction research in areas such as basic science [42], clinical therapeutics [91] and perhaps even drug design [33]. Our study greatly expands the breadth and diversity of early changes in the CXCL12/CXCR4 signaling network. We have shown through multiple independent means of validation that our dataset is consistent with what is currently understood about CXCL12/CXCR4 signaling. We confirmed associations with various signaling pathways that have already been described, e.g. T cell activation, EGF, and mTOR, and highlighted perhaps under-appreciated associations such as with microtubule dynamics. Our study also uncovered several phosphoproteins that may regulate cancer metastasis and HIV-1 infection of T cells, providing new avenues to expanding not only our basic understandings of these diseases but also to identify novel therapeutics.

## Materials and Methods

### CXCL12 treatment of CEM cells

CCRF-CEM cells (ATCC CCL-119) were cultured in RPMI-1640 with 10% (v/v) FBS, 100 units/mL of penicillin and 100 µg/mL streptomycin. For SILAC labeling, cells were cultured in RPMI lacking lysine or arginine (AthenaES) supplemented with 200 mg/L ^13^C_6_, ^15^N_4_ arginine, 40 mg/L ^13^C_6_, ^15^N_2_ lysine (Cambridge Isotopes) and 10% dialyzed FBS (Invitrogen 26400-036) for eight doublings. For proteomic experiments, cells were serum-starved overnight at a density of 1×10^6^ cells/mL in SILAC media. CXCL12 (Invitrogen PHC1346) stimulations (10 ng/mL final) were done in fresh serum-free SILAC media at 5×10^6^ cells/mL for 5 min. Cell suspensions were mixed with ice-cold PBS containing 1× phosphatase inhibitors (Sigma P5726, P0044) and centrifuged at 1500 RPM for 5 min at 4°C. Pellets were frozen on dry ice.

### Protein hydrolysis

Cell pellets were removed from storage at −80°C and placed on ice. Lysis buffer (0.3% SDS, 30 mM Tris, 20 mM HCl, pH 7.8, 3.8 mM MgCl_2_) with protease inhibitors (Sigma P2714) was used to disrupt the cell pellet (∼1 mL/50 uL cell pellet) and heated at 90°C for 5 min. The sample was incubated with 5 Units of benzonase (Novagen 70664-3) for 10 min at room temperature. Cysteines were alkylated by the addition of 50 mM iodoacetamide and kept in the dark for 30 min. Proteins were precipitated with 5 volumes of acetone and kept at −20°C for 2 hours to overnight. Protein precipitant was centrifuged (14,000× g, 15 min) and the pellet washed 2× with 80% acetone. The proteins were digested with 40 ug trypsin (Promega V511A) in 500 uL of 40 mM NH_4_HCO_3_, 0.1% Rapigest acid labile surfactant (Waters 186001861) at 37°C overnight. Before trypsin addition, protein content was measured using the Bradford assay. Rapigest was hydrolyzed with formic acid (2.5% v/v final concentration) for 1 hour at room temperature and centrifuged at 14,000× g for 20 min. Tryptic peptides were cleaned with a C18 Sep-Pak (Waters WAT036820) eluting with 2 mL 75%, 0.1% formic acid. Peptide concentrations were estimated by UV spectrophotometry @ 280 nm and equal amounts from isotopically heavy and light samples were mixed together.

### Hydrophilic interaction chromatography

The protocol of McNulty and Annan for phosphoproteome characterization utilizes hydrophilic interaction chromatography (HILIC) as a first dimension separation of tryptic peptides, the idea being that the more hydrophilic phosphopeptides are separated from the non-phosphopeptides thus facilitating capture by immobilized metal affinity chromatography (IMAC) with high selectivity [40]. HILIC was performed on a Beckman-Coulter System Gold HPLC with the following conditions: column, TSKgel Amide 80 4.6 mm×250 mm (Tosoh Biosciences); Buffers, A- 0.1% TFA in water, B- 90% CH_3_CN 0.1% TFA; Flow, 0.5 mL/minute; Equilibrate column 85% B 15% A, 1 to 2 mg. peptide in 0.5 mL 90% CH_3_CN 0.1% HCOOH loaded at 85% B for 10 min; Gradient, 85% to 70% B over 40 min, 70% to 10% B over 5 min, hold 10% B 5 min, return to 85% B over 2 min; Collect 2 min fractions from t = 5 to 65 min in 1 mL deepwell plate (Eppendorf C5096-0112). Ten percent of each fraction was reserved for whole proteome analysis and the remaining 90% of each fraction was used for phosphopeptide enrichment.

### Phosphopeptide Enrichment

Phosphopepitdes were enriched from the HILIC fractions using immobilized metal affinity chromatography in batch mode. Phos-Select Iron Affinity Beads (Sigma P9740) were added directly to the HILIC fractions (50 uL of 20% evenly suspended slurry) and mixed end over end for 30 min at room temperature. Fractions were transferred to 0.22 um centrifuge filter devices and centrifuged to remove the filtrate. Beads were washed with 300 uL 30% CH_3_CN, 250 mM AcOH, followed by a wash with water. Filtrates were discarded and phosphopeotdes eluted from the beads with 150 uL 400 mM NH_4_OH. After 10 min incubation, filtrates were recovered and lyophilized. Samples were reconstituted with 13 uL 0.1% HCOOH for analysis by mass spectrometry.

### Mass Spectrometry Analysis

Tryptic digests were analyzed on a hybrid LTQ Orbitrap mass spectrometer (Thermofisher Scientific, San Jose, CA) coupled with a NanoLC pump (Eksigent Technologies) and autosampler. Tryptic peptides were separated by reverse phase (RP)-HPLC on a nanocapillary column, 75 µm id×20 cm ProteoPep (New Objective, Woburn, MA, USA). Mobile phase A consisted of 1% methanol/0.1% formic acid and mobile phase B of 1% methanol/0.1% formic acid/79% acetonitrile. Peptides were eluted into the mass spectrometer at 300 nL/min with each RP-LC run comprising a 15 min sample load at 3% B and a 90 min linear gradient from 5 to 45% B. The mass spectrometer was set to repetitively scan m/z from 300 to 1800 (R = 100,000 for LTQ-Orbitrap) followed by data-dependent MS/MS scans on the six or ten most abundant ions, with a minimum signal of 1000, isolation width of 2.0, normalized collision energy of 28, and waveform injection and dynamic exclusion enabled. FTMS full scan AGC target value was 1e6, while MSn AGC was 5e3, respectively. FTMS full scan maximum fill time was 500 ms, while ion trap MSn fill time was 50 ms; microscans were set at one. FT preview mode, charge state screening, and monoisotopic precursor selection were all enabled with rejection of unassigned and 1+ charge states.

### Sequence database searching

Raw MS files were processed using MaxQuant (version 1.0.13.13) [92]. The .msm output files were searched against the International Protein Index human protein sequence database (version 3.52, concatenated with reversed decoy sequences and contaminants) using MASCOT search algorithm (Matrix Science, version 2.3). Fragment ion tolerance was set to 0.6 Da, with a maximum of one missed tryptic cleavage site. S-Carbamidomethyl cysteine was defined as a fixed modification while oxidized methionine, phospho-serine, phospho-threonine and phospho-tyrosine were selected as variable modifications. The false-discovery rate for peptides and proteins was set at 0.01. Reported phosphopeptide ratios were not used for calculating the protein ratios.

### Western blots

Cell pellets were dissolved in 1% triton x-100, 150 mM NaCl, 5 mM EDTA, phosphatase inhibitors (Sigma P5726, P0044) and a protease inhibitor cocktail (Roche 1836170) for 5 min on ice, then clarified by centrifugation at 20,000× g for 10 min. Lysates were denatured with LDS sample buffer (Invitrogen NP0007) with 2.5% (v/v) beta-mercaptoethanol and incubated at 70°C for 10 min. Samples were run on 10% Bis-Tris gels (Invitrogen NP0303) for 40 min at 200 V. Gels were transferred to PVDF membranes and blocked for 30 min in 5% (w/v) evaporated milk. Blots incubated at 4°C overnight with a 1∶1,000 dilution of primary antibodies. Antibodies against AKT (pS473), ERK1/2 (pT202/pY204), Stathmin (pS16), Stathmin, AKT1S1, α-tubulin, PBK (pT9), PLK1 (pT210) CDK1 (pY15), H3 (pS10), Rb (pS795), and Rb were obtained from Cell Signaling Technology. AKT1S1 (pT246) was obtained from Invitrogen and GAPDH from Calbiochem. HRP secondary antibodies (Jackson Labs) were used at 1∶20,000 for 30 min and antibodies for fluorescent blots, anti-rabbit 680 (Alexa, Invitrogen) and anti-mouse 800 (Rockland), were used at 200 ng/mL for 30 min.

### Pathway analysis and comparisons of datasets

Kyoto Encyclopedia of Genes and Genomes (KEGG) pathway analysis was done with DAVID [93], [94] (http://david.abcc.ncifcrf.gov) with default settings. Genes from all detected phosphopeptides served as a background. The fold-enrichment i.e., overlap, of a given dataset (dataset X) within CXCL12-responsive phosphosites or genes was determined with contingency tables by dividing the frequency of phosphosites or genes from dataset×within CXCL12-responsive phosphosites or genes by the frequency of phosphosites or genes from dataset×within CXCL12-nonresponsive phosphosites or genes. For comparisons with angiotensin phosphoproteomic data [44], were reanalyzed such that phosphopeptides at a given time post-stimulation were considered ‘hits’ if regulated 1.5-fold in both biological replicates. Cancer related genes [69] were obtained from the Wellcome Trust Sanger Institute Cancer Genome Project web site, http://www.sanger.ac.uk/genetics/CGP. HIV interactor genes were obtained from (http://www.ncbi.nlm.nih.gov/RefSeq/HIVInteractions).

### Statistical Analysis

Significance for KEGG cellular pathway enrichments were determined in DAVID [93], [94]. P-values for fold-enrichments between datasets were calculated with the one-sided Fisher's exact test (R version 2.12.0). All p-values were adjusted for multiple comparisons using the Benjamini and Hochberg method in R.

## Supporting Information

Table S1 Detailed information from MaxQuant searches of CEM phosphoproteome.
(XLS)

Table S2 Detailed information from MaxQuant searches of CXCL12-responsive phosphopeptides.
(XLS)
